# Economic Evaluation of a Problem Solving Intervention to Prevent Recurrent Sickness Absence in Workers with Common Mental Disorders

**DOI:** 10.1371/journal.pone.0071937

**Published:** 2013-08-12

**Authors:** Iris Arends, Ute Bültmann, Willem van Rhenen, Henk Groen, Jac J. L. van der Klink

**Affiliations:** 1 Department of Health Sciences, Community and Occupational Medicine, University Medical Center Groningen, University of Groningen, Groningen, the Netherlands; 2 Center for Human Resource, Organization and Management Effectiveness, Business University Nyenrode, Breukelen, the Netherlands; 3 Occupational Health Services, Utrecht, the Netherlands; 4 Department of Epidemiology, University of Groningen, University Medical Center Groningen, Groningen, the Netherlands; Catholic University of Sacred Heart of Rome, Italy

## Abstract

**Objectives:**

Workers with common mental disorders (CMDs) frequently experience recurrent sickness absence but scientifically evaluated interventions to prevent recurrences are lacking. The objectives of this study are to evaluate the cost-effectiveness and cost-benefit of a problem solving intervention aimed at preventing recurrent sickness absence in workers with CMDs compared to care as usual.

**Methods:**

An economic evaluation was conducted alongside a cluster-randomised controlled trial with 12 months follow-up. Treatment providers were randomised to either a 2-day training in the SHARP-at work intervention, i.e. a problem solving intervention, or care as usual. Effect outcomes were the incidence of recurrent sickness absence and time to recurrent sickness absence. Self-reported health care utilisation was measured by questionnaires. A cost-effectiveness analysis (CEA) from the societal perspective and a cost-benefit analysis (CBA) from the employer’s perspective were conducted.

**Results:**

The CEA showed that the SHARP-at work intervention was more effective but also more expensive than care as usual. The CBA revealed that employer’s occupational health care costs were significantly higher in the intervention group compared to care as usual. Overall, the SHARP-at work intervention showed no economic benefit compared to care as usual.

**Conclusions:**

As implementation of the SHARP-at work intervention might require additional investments, health care policy makers need to decide if these investments are worthwhile considering the results that can be accomplished in reducing recurrent sickness absence.

## Introduction

The costs of mental disorders to society are substantial in terms of medical care consumption, but even more because of productivity loss due to sickness absence, work disability and at-the-job productivity loss [1–7]. Common mental disorders (CMDs, i.e. depressive, anxiety and adjustment disorders), as opposed to severe mental disorders, account for the majority of costs related to mental ill-health [7]. However, evidence for work-related interventions is much more established for severe mental disorders, such as supported employment programs [8]. In the last decade, several studies have focused on workers suffering from CMDs and have evaluated interventions to enhance return to work (RTW) [9–12]. In these studies, RTW has been defined as endpoint while recent research has shown that 20% to 30% of the workers who returned to work after sickness absence due to CMDs experience recurrent sickness absence [13,14]. Moreover, recurrent sickness absence is often more serious and long-lasting than the initial sickness episode due to CMDs [13]. Thus, more attention is needed for enhancing sustainable RTW of workers with CMDs by preventing recurrent sickness absence.

The SHARP-at work intervention is developed to prevent recurrent sickness absence in workers who returned to work after sickness absence due to CMDs [15]. The intervention consists of problem-solving treatment provided by occupational physicians (OPs). OPs guide workers through a problem-solving process focused on establishing solutions for problems and opportunities encountered when back at work. Furthermore, consultations between the worker and the supervisor are stimulated by the OP to achieve solutions that can be readily implemented. The intervention was compared to care as usual (CAU) in a cluster-randomised controlled trial (cluster-RCT) and has shown to be effective in reducing recurrent sickness absence (Arends et al., submitted).

Before implementing the SHARP-at work intervention in the occupational health care practice, insight is needed in the relationship between intervention costs and benefits compared to CAU [16]. Therefore, the objective of this study was to perform an economic evaluation of the SHARP-at work intervention compared to CAU. Cost-effectiveness was evaluated from the societal perspective and cost-benefit from the societal and employer perspective.

## Methods

### Study design

An economic evaluation was conducted alongside a cluster-RCT. OPs, who conducted the intervention, were recruited through 365/ArboNed, one of the largest Occupational Health Services in the Netherlands. The Medical Ethical Board of the University Medical Center Groningen provided approval for the study design, the research protocol, questionnaires, information letters and the informed consent. The trial was registered in the Dutch Trial Register (NTR) with registration number NTR1963. More detailed information on the study design and procedure of the cluster-RCT has been presented elsewhere [15].

### Study setting

In the Netherlands, the employer pays sickness absence benefits to the sick-listed worker for two years. During these two years, both the employer and sick-listed worker are responsible for RTW. The employer is obliged to contract an OP to help guiding the RTW process. If RTW has not been accomplished after two years, the Social Security Agency (SSA) evaluates if sufficient RTW efforts have been made by the employer and worker and decides on the percentage of work disability for which the worker will be compensated by the SSA. Costs for treatment and work accommodations are the responsibility of the employer, but compensation can be requested from the SSA for work accommodations.

### Study population

Participants were recruited by OPs between January 2010 and June 2011. Eligible participants were workers between 18 and 63 years, had to be diagnosed with a CMD by their OP at the start of sickness absence and had to be ready to (partially or fully) RTW. A detailed overview of all inclusion and exclusion criteria has been presented elsewhere [15].

### Randomisation and blinding

OPs were randomised to the intervention or control group based on a computer-generated random allocation sequence because workers could not be randomly allocated to OPs as OPs are bound to companies. Since a worker’s allocation was predefined based on the OP’s allocation, we only provided information on the treatment the worker would receive and blinded the worker for study design and comparison group. OPs were not blinded for study design and allocation.

### Interventions

#### SHARP-at work intervention

A detailed description of the intervention has been provided elsewhere (Arends et al., submitted for publication). In brief, the SHARP-at work intervention was developed to prevent recurrent sickness absence by structured OP guidance after RTW. The intervention was started by OPs when participants on sickness absence due to CMDs were ready to RTW. Five steps had to be followed by the participant when RTW was started. The OP monitored that all steps were taken and activated the participant when needed. For each step, facilitating assignments for the worker were at the OP’s disposal. The five steps comprised: (1) making an inventory of problems and/or opportunities encountered at work after RTW, (2) brainstorming about solutions/realisations, (3) writing down solutions/realisations and the support needed and assessing the applicability of these solutions, (4) discussing solutions/realisations and making an action plan with the supervisor, and (5) evaluating the action plan/implementation of solutions. Two to five consultations, of 30 minutes each, were recommended to OPs. The first of five assignments (i.e. making an inventory of problems and opportunities and assessing the help needed to solve them) instigated the problem solving process and was therefore a key element. OPs received a two-day training in the SHARP-at work intervention and had three feedback moments to discuss their experiences with conducting the intervention.

#### Care as usual

All participating OPs were already trained in the evidence-based guideline of the Netherlands Society of Occupational Medicine “The treatment of workers with mental health problems by the OP” [17,18]. The guideline is primarily directed at structuring OP’s treatment to help sick-listed workers with mental health problems to RTW. Though one consultation has to take place after RTW to address relapse prevention, limited attention is given to a structured follow-up of OP’s treatment after RTW has been accomplished.

### Economic evaluation

An economic evaluation was performed from the societal and employer perspective. The evaluation from the societal perspective consisted of a cost-effectiveness analysis (CEA) with the difference in incidence of recurrent sickness absence and time to recurrent sickness absence between the two study groups as the outcomes. Costs associated with health care utilisation (including the costs for the intervention or CAU) were included in the CEA. From the employer perspective, a cost-benefit analysis (CBA) was performed comparing the two study groups regarding costs associated with health care utilisation (including the costs for the intervention or CAU) and the monetary value of differences in productivity.

#### Effects

The effect measures for the CEA were incidence of recurrent sickness absence and time to recurrent sickness absence during 12 months follow-up. Recurrent sickness absence was defined as ≥ 30% decrease in working days per week due to sickness absence, regardless of partial or full RTW. Recurrent sickness absence days were corrected for partial RTW by dividing the sickness absence days by 1/RTW percentage.

#### Health care costs

Data were collected using the Trimbos/iMTA questionnaire for Costs associated with Psychiatric Illness (TiC–P) with a 4-week recall period at baseline and at 3, 6 and 12 months follow-up. The data were linearly interpolated over 12 months. The unit prices used for valuing resource utilisation are presented in Table 1. The study’s index year was 2009. The Dutch Manual for Costing was used for calculating standard prices [19]. Costs for alternative care were based on real costs reported by the participants. Medication costs were valued with cost prices of the Royal Dutch Society for Pharmacy [20]. Two questions on number of consultations with the OP and company social worker were added to the Tic-P to collect data on use of occupational health services. For the calculation of costs from the societal perspective, the OP consultations were valued using the cost level for general practitioners, and consultations with the company social worker were valued using the cost level for social workers. For the calculation of costs from the employer perspective real employer prices for OPs and company social workers were used which were provided by the Occupational Health Service.

**Table 1:** Unit prices used and mean (SD) total costs per study group.

		**Mean costs (SD)**	
**Type of costs**	**Unit prices**	**SHARP**	**CAU**
Health care costs for society			
General practitioner	29^a^	59 (63)	61 (70)
Regional Institute for Community Mental Health Care	70^a^	69 (140)	96 (194)
Psychiatrist	107^a^	22 (74)	67 (212)
Psychologist	83^a^	212 (228)	209 (205)
Occupational physician	29	81 (48)	60 (46)
Company social worker	68	34 (80)	16 (48)
Medical specialist	75^a^	103 (239)	91 (208)
Physiotherapist	37^a^	61 (138)	68 (118)
Social worker	68^a^	15 (52)	15 (80)
Alternative health care	31-64^c^	66 (137)	91 (189)
Psychiatric part-time or day program	200^a^	48 (347)	27 (186)
Hospitalisation	452-597^a^	164 (1000)	42 (163)
Prescribed medication	Variable^b^	43 (83)	38 (65)
Self-purchased medication	Variable^c^	33 (105)	80 (158)
Out-of-pocket costs	Variable^c^	29 (109)	38 (159)
SHARP-at work intervention	661	661	0
Total health care costs		4167 (9407)	2403 (2360)
**Costs of occupational health services for employer**			
Occupational physician	154	420 (250)	314 (240)
Company social worker	121	248 (239)	178 (125)
SHARP-at work intervention	661	661	0
Total costs of occupational health services		1143 (342)	343 (254)
**Costs of productivity loss**			
Productivity loss net HCA			
Only sickness absence		37265 (26227)	32019 (22442)
Combined^d^		36072 (20015)	31342 (24039)
Productivity loss net FCA			
Only sickness absence		27789 (17185)	24594 (15993)
Combined^d^		28194 (14529)	24264 (18069)

All costs are given in euros. SHARP = intervention group; CAU = care as usual; HCA = human capital approach; FCA = friction cost approach.

a Price according to Dutch guidelines for costing studies.

b Price according to the Royal Dutch Society for Pharmacy

c Price according to self-report of participants

d Productivity loss costs are a combination of sickness absence costs and costs due to lost productivity at work (based on N=35 in SHARP group and N=37 in CAU group).

#### Productivity loss

For the CBA, productivity loss was operationalized as costs resulting from sickness absence and at-work productivity loss (i.e. presenteeism). To measure sickness absence costs, administrative data were collected on cumulative number of days of sickness absence over a period of 12 months. Calendar days of sickness absence were corrected for part-time sickness absence and converted to number of working hours based on participants’' work contract. For the calculation of productivity loss costs, we assumed that participants were 100% productive during the hours of work resumption. At-work productivity loss was assessed with one question of the TiC–P stating: "How many extra hours would you have to work to catch up on tasks you were unable to complete in normal working hours due to health problems over the past two weeks?" Sickness absence costs and costs for at-work productivity loss were calculated by multiplying the number of sickness absence hours by the estimated cost of production loss for a worker per hour of absence, differentiating between costs for men and women. We used the Human Capital Approach (HCA) and the Friction Cost Approach (FCA) to calculate the total costs of production loss. A friction period of 154 days and an elasticity of 0.8 were applied in the FCA [19,21].

### Data analysis

The economic evaluation was performed according to the intention-to-treat principle. Discounting of costs was not applied because the follow-up was limited to one year. Sickness absence data were collected for 145 (92%) participants and a complete follow-up on self-reported data was available for 99 (63%) participants.

For the CEA, the incremental cost-effectiveness ratio (ICER) was calculated by dividing the incremental costs by the incremental effects. The incremental costs consisted of the difference in all health care utilisation costs (including the intervention costs) between the intervention and control group. Two incremental effect measures were calculated: (1) the difference in incidence of recurrent sickness absence and (2) the difference in time (measured in days) to recurrent sickness absence between the intervention and control group. The ICER represents the additional investments needed to prevent one case of recurrent sickness absence or to prevent one day of recurrent sickness absence. For the CBA from the employer’s perspective, the net monetary benefit (NMB) was calculated by subtracting the difference in costs for occupational health services (including the intervention costs) between the intervention and control group from the difference in costs of productivity loss between the two groups. Total costs of productivity loss was calculated with and without costs due lost productivity at work, next to costs of sickness absence, as data on lost productivity at work were only available for 51% of the study sample. The CBA was performed using both the HCA and FCA. The mean difference in costs and benefits between the intervention and control group and the 95% confidence intervals (CI) were calculated with multilevel regression analysis to account for the study’s multilevel design (i.e. adjusting for the influence at the OP-level as participants were nested in OPs).

The 95% CIs for the incremental costs were estimated using a bias corrected and accelerated bootstrapping procedure with 5000 replications [22]. Bootstrapped cost-effect pairs were plotted on a cost-effectiveness plane. Cost-effectiveness acceptability curves were generated if the ICER was located in the north-east quadrant [23]. Sensitivity analysis for the CEA was conducted to assess the effect of one extreme outlier. Sensitivity analyses for the CEA and CBA were conducted to assess the effect of reducing the intervention costs to €30 per participant. This reduction in costs was calculated under the assumption that, in practice, OPs will treat more workers according to the intervention than the 80 workers that were included in the intervention group of the present study. In this way, the training costs of the intervention could be divided over more workers causing a reduction in intervention costs per worker. Based on the OHS’s data, every OP treats 2500 to 3000 workers of which 32 to 39 experience sickness absence due to CMDs within one year [15]. Taking the conservative assumption that OPs can treat 24 of the 32 to 39 workers according to the intervention per year, the intervention training costs for the 73 OPs that participated in the study can be divided over 1752 (73 x 24) workers, leading to a total amount of €30 per worker instead of €661 (see also Table 2). Data processing was performed in SPSS 20.0. Calculation of CIs and CEA analyses were conducted in R [24].

**Table 2:** Costs of the SHARP-at work intervention.

**Resources**	**Description**	**Aggregated costs in euros**
Costs for training OPs in the intervention		
Trainer costs^1^	Preparation of training: 2 trainers, 2-10 hours, €100 per hour	1200
	Training sessions: 2 trainers, 12-108 hours, €100 per hour	12000
	Follow-up meetings: 1 trainer, 6 hours, €100 per hour	600
OP attendance costs^2^	Training of OPs: 73 OPs, 12 hours, €40 per hour	35040
	Follow-up meetings: 40 OPs, 1,5 hours, €40 per hour	2400
Additional training costs	Rent for training location, refreshments and study materials^2^	1660
Total training costs	Sum of trainer costs, OP attendance costs and additional training costs	52900
Training costs per worker	Total training costs divided by 80 workers	661
Training costs per worker sensitivity analysis	Total training costs divided by 1740 workers	30

OP = occupational physician

1 Based on price requested by trainer.

2 Based on OP’s mean wage paid by the Occupational Health Service that was responsible for training the OPs.

## Results

### Participants

OPs recruited 212 workers of whom 158 agreed to participate. Eighty participants were treated by OPs in the intervention group and received the SHARP-at work intervention, and 78 participants were treated by OPs in the control group and received CAU. Baseline characteristics of the study population are presented in Table 3.

**Table 3:** Baseline characteristics of the study population.

				**SHARP** (n = 80)			**CAU** (n = 78)	
**Characteristics**				M / n	SD / %		M / n	SD / %
Socio-demographic characteristics								
Age (years)				41.3	9.4		43.3	9.8
Gender (male)				27	33.8		38	48.7
Marital status (married or living together)				67	83.8		60	76.9
Breadwinner (yes)				40	50.0		49	62.8
Educational level								
	Low			6	7.5		13	16.7
	Intermediate			36	45.0		40	51.3
	High			38	47.5		23	29.5
Clinical characteristics								
ICD diagnosis by OP								
	F32.9 Depressive episode, unspecified			4	5.0		12	15.4
	F41.9 Anxiety disorder, unspecified			0	0.0		2	2.6
	F43.2 Adjustment disorders			58	72.5		39	50.0
	F43.9 Reaction to severe stress, unspecified			1	1.25		0	0.0
	R45 Symptoms and signs involving emotional state			7	8.75		14	17.9
	Z73.0 Burn-out			2	2.5		7	9.0
	Other			8	10.0		4	5.1
Work-related characteristics								
Type of occupation								
	Commercial service providers			23	28.8		11	14.1
	Management			11	13.8		15	19.2
	Administrative staff			19	23.8		12	15.4
	ICT staff			4	5.0		4	5.1
	Sales staff			2	2.5		5	6.4
	Health care providers			12	15.0		12	15.4
	Hotel and catering staff			3	3.8		0	0.0
	Stock and/or transport staff			1	1.3		11	14.1
	Designers/planners			3	3.8		2	2.6
	Mechanics/repairmen			2	2.5		5	6.4
Employment (hours per week)				32.6	7.0		32.9	7.3
Irregular work (e.g. shift work)				6	7.5		10	12.8
Executive/manager responsibilities				23	28.8		21	26.9
Duration of sickness absence				130.9	94.2		99.3	66.1
WRFQ-Total score				66.9	15.5		61.0	20.0
Health-related characteristics								
4DSQ			Distress	13.8	7.5		15.5	7.5
			Depression	1.5	2.1		2.0	2.4
			Anxiety	3.1	3.3		3.6	3.5
			Somatization	7.9	5.3		7.9	5.5
HADS			Depression	7.0	4.5		7.3	4.4
			Anxiety	7.2	3.9		7.8	3.4

SHARP = intervention group; CAU = care as usual group; OP = occupational physician; WRFQ = Work Role Functioning Questionnaire; 4DSQ = Four-Dimensional Symptom Questionnaire; HADS = Hospital Anxiety and Depression, Scales; M = mean; SD = standard differentiation

### Effects on recurrent sickness absence

The incidence of recurrent sickness absence during 12 months follow-up was 39% for the SHARP group and 62% for the CAU group. Based on the bootstrap procedure, the mean effect difference between the SHARP and CAU group was 24% (95% CI 3% to 45%) in favour of the SHARP group (Table 4). The median number of days to recurrent sickness absence was 365 (inter quartile range (IQR) 174 to 365) in the SHARP group (i.e. 50% of the participants in the SHARP group did not have a recurrent sickness absence episode within the 12 months follow-up) and 253 (IQR 117 to 365) in the CAU group. The mean effect difference between the SHARP and CAU group was 55 (95% CI 2.85 to 106.09) days in favour of the SHARP group, i.e. the SHARP group experienced recurrent sickness absence 55 days later than the CAU group (Table 4).

**Table 4:** Mean cost and effect differences between the SHARP and CAU group.

Analysis^a^			ΔC (95% CI)		ΔE (95% CI)		
		euros			percentage/days/euros^d^	ICER	NMB^e^
Total group							
	CEA-incidence of RSA	1932 (-318 to 5350)			0.24 (0.03 to 0.45)	10605	
	CEA-time to RSA	1358 (-945 to 4886)			55 (2.85 to 106.09)	2183	
	CBA HCA only sickness absence	800 (678 to 922)			5246 (-2701 to 13192)		6046
	CBA FCA only sickness absence	800 (678 to 922)			3195 (-2214 to 8604)		3995
	CBA HCA combined^b^	800 (678 to 922)			4730 (-5699 to 15158)		5530
	CBA FCA combined^b^	800 (678 to 922)			3929 (-3764 to 11623)		4729
Excluding outlier^c^							
	CEA-incidence of RSA	-133 (-1155 to 914)			0.25 (0.03 to 0.46)	-533	
	CEA-time to RSA	-129 (-1266 to 964)			59 (5.95 to 111.15)	-2	

CEA = cost effectiveness analysis; RSA = recurrent sickness absence; CBA = cost benefit analysis; ΔC = mean cost difference; ΔE = mean effect difference; HCA = human capital approach; FCA = friction cost approach; ICER = incremental cost effect ration; NMB = net monetary benefit

a In the CEA, ΔC is the mean difference in total health care costs and ΔE is the mean difference in percentage of workers that experienced recurrent sickness absence; in the CBA, ΔC is the mean difference in total occupational health care costs, including the intervention, for the employer and ΔE is the mean difference in sickness absence costs estimated by the HCA or FCA.

b Productivity loss costs are a combination of sickness absence costs and costs due to lost productivity at work.

c Sensitivity analysis excluding one extreme outlier.

d Differences in CEA effects are presented in (1) percentage of workers that experienced recurrent sickness absence, (2) number of days to recurrent sickness absence; differences in CBA effects are presented as costs in euros.

e Negative values of the NMB imply lower costs for the intervention group compared to the control group.

### Health care and productivity loss costs

The mean costs of health care utilisation are presented in Table 2. An important cost driver was care by the psychologist. There were small differences between the SHARP and CAU group regarding non-occupational health care use. The SHARP group more frequently visited the OP and company social worker. Following this, total occupational health care costs for the employer were significantly higher in the SHARP group (Table 4). The costs of the SHARP-at work intervention were €661 per worker. We also calculated the intervention costs based on the assumption that, in practice, every OP could treat at least 24 workers according to the intervention lowering intervention costs per worker to €30 (Table 2). The difference in mean total health care costs between the two study groups was mainly due to one outlier in the SHARP group whose total health care costs were more than nine times higher than the upper limit of the 95% CI of the total health care costs of the SHARP group. The high costs for this outlier were mainly due to hospitalisation in a psychiatric ward.

No significant differences were found between the SHARP and CAU group regarding cost of productivity loss (Table 4). For both groups, cost of productivity loss represented 87% to 93% of the total costs, depending on how it was measured (HCA or FCA).

### Cost-effectiveness analysis

**Figure 1:**
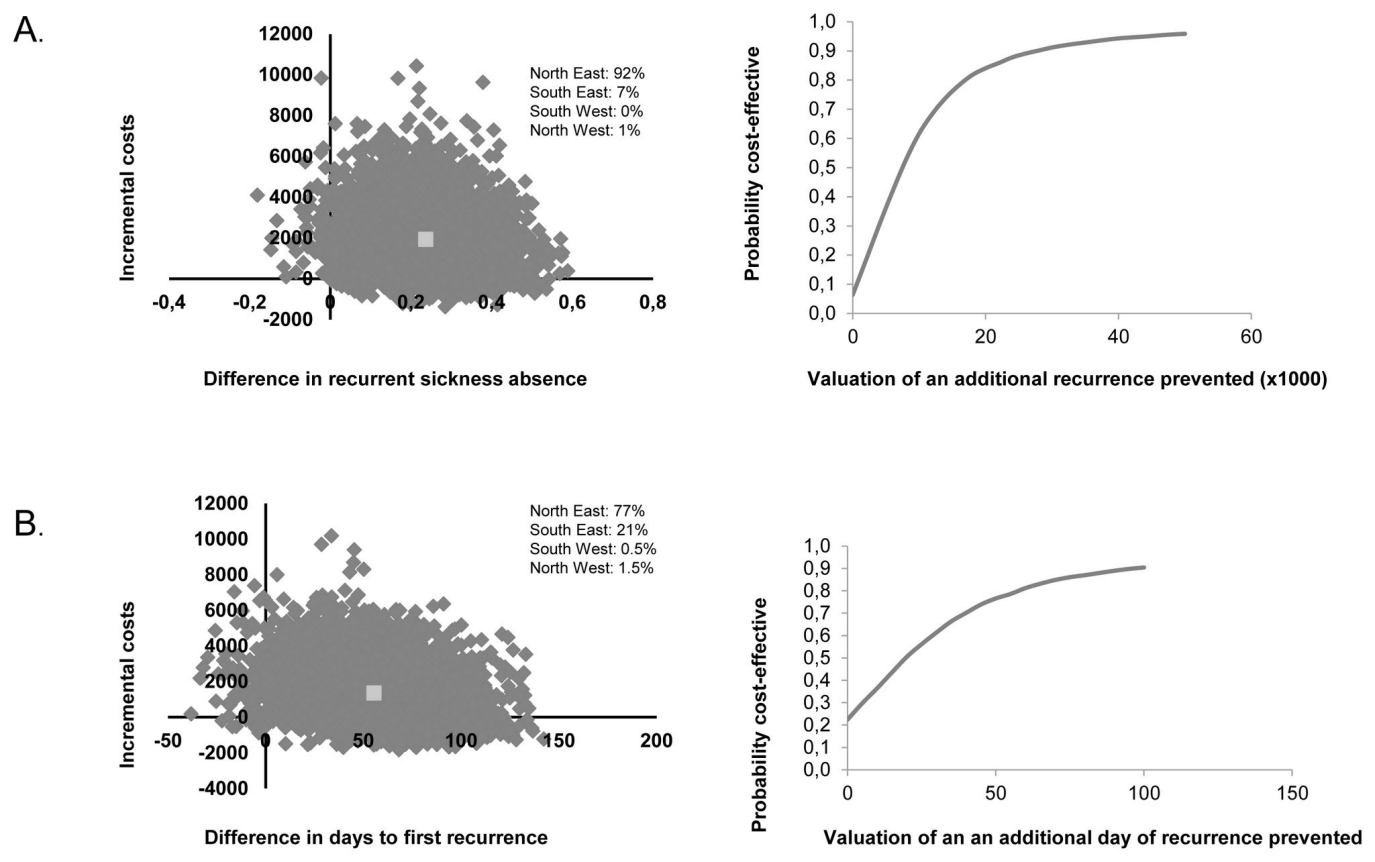
Cost-effectiveness planes and acceptability curves for (A) the difference in incidence of recurrent sickness absence and (B) time to recurrent sickness absence.

The CEA with incidence of recurrent sickness absence as effect measure showed an ICER of €10.605 per percent of prevented recurrent sickness absence episode, i.e. an additional €10.605 were needed in the SHARP group to have 1% less recurrent sickness absence compared to the CAU group (Table 4). The cost-effectiveness plane showed that 92% of the bootstrap cost-effectiveness pairs were in the north-east quadrant (Figure 1A). The cost-effectiveness acceptability curve showed that if one is willing to invest €20.000 for 1% less recurrent sickness absence, there is a 84% probability that the intervention is cost-effective compared to CAU (Figure 1A). The CEA with time to recurrent sickness absence as effect measure showed an ICER of €2813 per one day of prevented recurrent sickness absence, meaning that an additional €2813 was needed in the SHARP group to prevent one day of recurrent sickness absence. The cost-effectiveness plane showed that 77% of the bootstrap cost-effectiveness pairs were in the north-east quadrant (Figure 1B). The cost-effectiveness acceptability curve showed that if one is willing to invest €70 to prevent one day of recurrent sickness absence, there is a 85% probability that the intervention is cost-effective compared to CAU (Figure 1B). Thus, the SHARP-at work intervention was more effective but also more costly compared to CAU.

**Figure 2:**
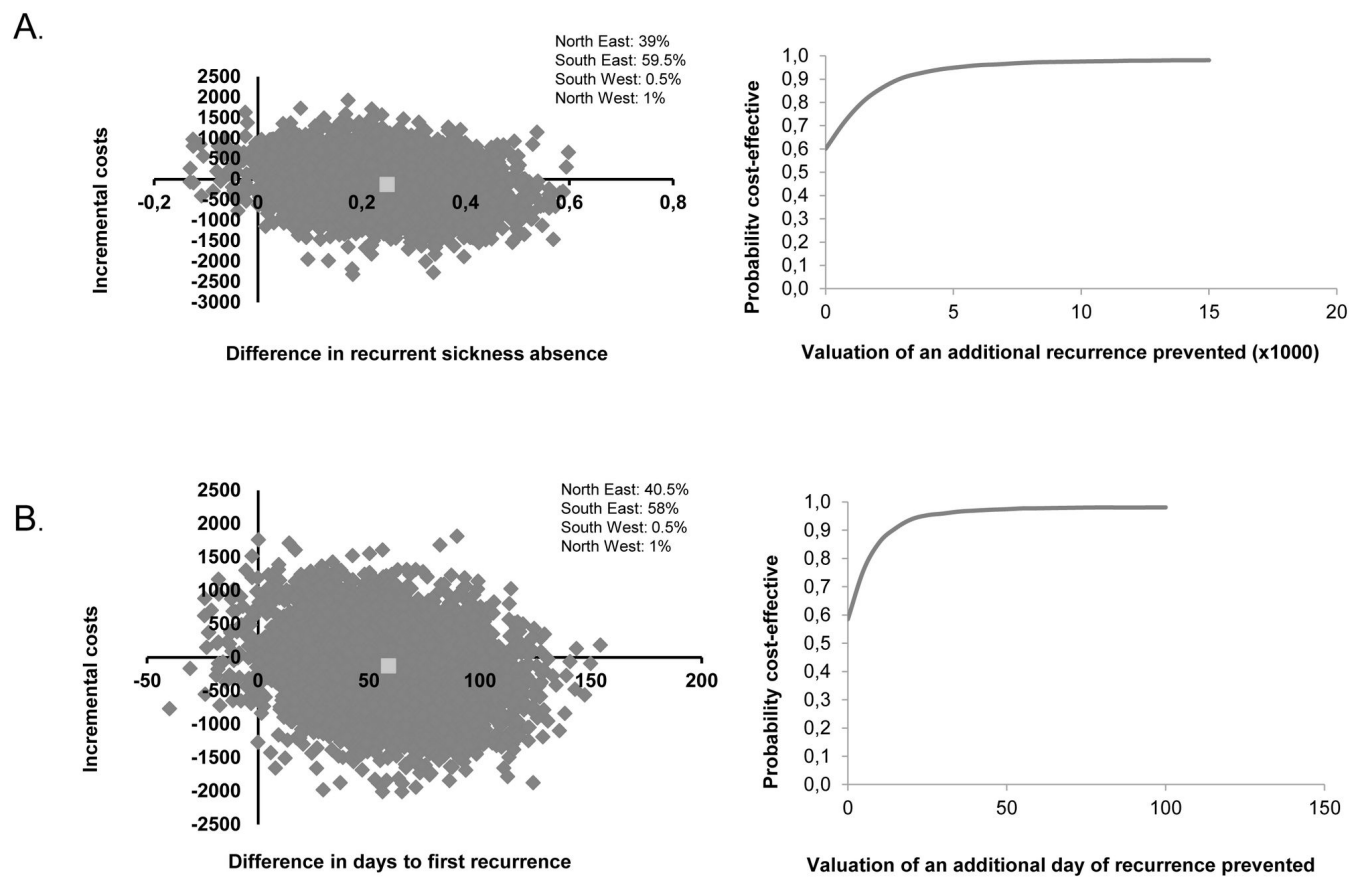
Cost-effectiveness planes and acceptability curves excluding one outlier for (A) the difference in incidence of recurrent sickness absence and (B) time to recurrent sickness absence.

The sensitivity analysis excluding the outlier showed an ICER of €-533 for the incidence of recurrent sickness absence, indicating that the intervention was cost-effective: 1% less recurrent sickness absence saved €533. In the cost-effectiveness plane 60% of the bootstrap cost-effectiveness pairs were in the south-east quadrant (Figure 2A). The cost-effectiveness acceptability curve showed a 98% probability that the intervention is cost-effective compared to CAU if one is willing to invest €15.000 for 1% less recurrent sickness absence (Figure 2A). Similarly, the sensitivity analysis excluding the outlier changed the direction of the primary results regarding time to recurrent sickness absence. An ICER of €-2 was found, indicating that the intervention was cost-effective compared to care as usual: the prevention of one day of recurrent sickness absence saved €2. The cost-effectiveness plane showed that 58% of the bootstrap cost-effectiveness pairs are in the south-east quadrant (Figure 2B). The cost-effectiveness acceptability curve showed a 98% probability that the intervention is cost-effective compared to CAU if one is willing to invest €70 to prevent one day of recurrent sickness absence (Figure 2B).

Sensitivity analyses with reduced intervention costs did not change the direction of the primary analyses.

### Cost-benefit analysis

The CBA from the employer’s perspective showed that the mean cost difference for occupational health services was in favour of the CAU group. The mean costs were €800 (95% CI 678 to 922) higher in the SHARP group compared to the CAU group. The mean cost difference for productivity loss was also in favour of the CAU group. According to the HCA, only including sickness absence costs, the mean costs for productivity loss were €5246 (95% CI -2701 to 13192) higher in the SHARP group. Following the FCA, only including sickness absence costs, the mean costs for productivity loss were €3195 (95% CI -2214 to 8604) higher in the SHARP group. Thus, no net monetary benefit was achieved with the SHARP-at work intervention compared to CAU. The sensitivity analyses with reduced intervention costs did not change these results.

## Discussion

The SHARP- at work intervention had a superior effect on the incidence of and time to recurrent sickness absence but had no economic benefit compared to care as usual. From a societal perspective, there were no significant differences in health care costs between the SHARP group and CAU group. Employer costs for occupational health care were significantly higher in the SHARP group compared to CAU. Costs due to lost productivity did not significantly differ between the two study groups. Thus, to realise the effect on recurrent sickness absence, additional monetary investments in the SHARP-at work intervention were needed. Even though an economic benefit of the intervention was not found, a societal benefit may be realised when the reduction in recurrent sickness absence results in more stable work participation.

Sensitivity analyses for the CEA excluding one major outlier changed the direction of the primary CEA results. Excluding the outlier, the SHARP-at work intervention was cost-effective in preventing the incidence of recurrent sickness absence and increasing time to recurrent sickness absence.

Although the SHARP-at work intervention was effective in reducing the incidence of recurrent sickness absence and increased the time to recurrent sickness absence, the CBA showed no effect on reduced costs of productivity loss. This result might be counterintuitive as a reduced incidence of recurrent sickness absence would be expected to result in reduced sickness absence days and, thus, reduced costs due to productivity loss. However, costs due to productivity loss were found to be somewhat higher for the SHARP group, meaning that the SHARP group had more sickness absence days. This result may be partly explained by the fact that the CAU group had a shorter duration of sickness absence and a higher RTW percentage at baseline, resulting in less sickness absence days. Possibly, the study’s one-year follow-up timeframe was too short to pick up long-term effects on sickness absence.

No previous studies have been published on an economic evaluation of an intervention to prevent recurrent sickness absence in workers who returned to work after sickness absence due to CMDs. Recently, several studies were published on economic evaluations of RTW interventions for workers with mental health problems [25–28]. Comparing our study results with these RTW studies is complicated as the study populations and the effect measures differ. The participants in the RTW studies were still off work and the interventions aimed to facilitate RTW. Effect measures in the economic evaluations of these RTW studies are focused on days to first or full RTW. Most of the RTW studies showed no economic benefit of the RTW intervention under investigation [25,27,28].

### Strengths and limitations

The strengths of this study are the pragmatic design, data collection on productivity loss and the use of a societal and employer perspective for the economic evaluation. Firstly, the pragmatic study design enabled an economic evaluation of the SHARP-at work intervention in a real life situation and in a heterogeneous study population. Participants lived in different parts of the Netherlands, worked for small and large companies in different branches and occupied various job positions. This increased the external validity of the study results. Secondly, data on productivity loss included costs due to self-reported lost productivity at work, i.e. presenteeism, next to costs due to sickness absence. Even though information on productivity loss at work was only collected for 51% of the study sample, the CBA including this information gives a clear indication of the underestimation of productivity loss costs when only using sickness absence data and assuming that participants are 100% productive when at work. As presenteeism seems to be an important contributor to productivity loss among workers with mental health problems, this is an important variable to include in economic evaluations [6,29,30]. However, previous studies on the economic benefit of occupational health care interventions for workers with mental health problems often missed information on presenteeism [26–28]. Lastly, the cost-effectiveness evaluation from the broad societal perspective increased the generalisability of the results, while the cost-benefit analysis from the employer perspective provided a realistic perspective on the distribution of costs and benefits of the SHARP-at work intervention within the Dutch social security context.

Some methodological limitations need to be considered. Data on health care utilisation were collected based on retrospective, self-reported questionnaires which may have biased the results. Although participants received diaries to keep track of health care utilisation to improve the reliability of the self-reported questionnaires, these diaries were sent for participants’ own convenience and not recollected. Thus, we were not able to check whether the diaries were used. Furthermore, data on health care utilisation during the past month were only collected at four measurement points and were linearly interpolated to 12 months, assuming a linear time trend in health care utilisation. Health care costs may have been overestimated or underestimated if health care utilisation between two time measurements was not linear over time. However, this probably will not have affected the direction of our results, as the health care costs only presented a small proportion of the total costs and data were linearly interpolated for both study groups. Another limitation is the missing data due to loss to follow-up: 62% of the participants had complete cost data. By using a bootstrap procedure with 5000 replications for the CEA, the problem of missing data was partly averted. Furthermore, we chose to conduct complete case analysis because we assumed that the data was missing completely at random: no significant differences were found between participants who were lost to follow-up and those who completed the study (Arends et al., submitted for publication). One exception might have been the data collected on productivity loss at work. Many participants responded to this question with a question mark, indicating that participants had trouble understanding this item. Therefore, we decided to also conduct the CBA excluding the information on lost productivity at work. As 62% of the data could be used for the CEA and CBA and no power calculation was conducted for the economic evaluation, it could be possible that the study was underpowered. For example, sensitivity analyses excluding one major outlier in the intervention group showed that the CEA results were strongly influenced by this outlier as the direction of the results changed after excluding the outlier. It should also be acknowledged that using the wage costs of hours off work and hours not being productive while at work may not be the best measures for assessing productivity loss. Productivity loss also depends on the level of teamwork, the time sensitivity of output and the replaceability of a worker. Finally, no information was collected on the costs of workplace adaptations that possibly resulted from the SHARP-at work intervention. This might have caused an underestimation of the intervention costs. However, costs due to possible workplace adaptations would be difficult to measure in a population of workers with CMDs. These workers do not so much require adaptations in workplace equipment or design but are more in need of, for example, frequent/longer breaks, lower work pace or other job content [31] of which the costs are hard to estimate. Comparable workplace adaptations could also have been introduced in the CAU group, as participants in this group also had consultations with their OPs. Thus, differences in total health care costs between the two study groups would probably not drastically increase by including costs related to workplace adaptations.

## Conclusion

The SHARP-at work intervention is effective in reducing recurrent sickness absence and in increasing time to recurrent sickness absence but is associated with higher costs compared to CAU. Bearing in mind the study’s limitations, future research needs to confirm that the SHARP-at work intervention is not cost-effective and cost-beneficial. As implementation of the SHARP-at work intervention might require additional investments, health care policy makers need to decide if these investments are worthwhile considering the results that can be accomplished in reducing recurrent sickness absence.
